# Profil anatomopathologique du cancer du sein dans le cap bon tunisien

**DOI:** 10.11604/pamj.2017.26.11.11382

**Published:** 2017-01-05

**Authors:** Ghada Sahraoui, Fatma Khanchel, Emna Chelbi

**Affiliations:** 1 Service d’Anatomie et Cytologie Pathologiques, Hôpital Mohamed Taher Mâamouri, Nabeul, Tunisie

**Keywords:** Carcinome mammaire, histopathologie, Tunisie, Breast cancer, histopathology, Tunisia

## Abstract

Le cancer du sein est le cancer le plus fréquent de la femme en Tunisie et dans le monde. Dans le Cap Bon tunisien, les particularités anatomopathologiques de ce cancer n'ont pas été précisées auparavant. Leur connaissance est nécessaire pour l'adaptation des systèmes de prévention et de soins dans la région. Le but de notre étude était de déterminer le profil anatomopathologique des carcinomes mammaires dans l'unique laboratoire d'anatomie pathologique publique de la région. Il s'agissait d'une étude descriptive rétrospective des carcinomes mammaires diagnostiqués chez 116 malades dans notre laboratoire sur une période de 5 ans de Juillet 2010 à Juillet 2015. Notre étude a inclus 116 patientes. L'âge moyen était de 51 ans. La taille tumorale histologique moyenne était de 31 mm. Le diagnostic initial était posé sur pièce de tumorectomie dans 83% des cas. Le carcinome infiltrant de type non spécifique était le type histologique le plus fréquent. Le grade SBR III était majoritaire. L'invasion lympho-vasculaire était présente dans 33% des cas. Le curage axillaire était positif dans 72% des cas. Les récepteurs hormonaux étaient positifs dans 73% des cas. Les récepteurs de l'Her2-Neu étaient surexprimés dans 19% des cas. Le ki67 était ≥ 14% dans 38%. Le sous-type moléculaire le plus fréquent était le luminal A. Le carcinome mammaire dans la région du Cap Bon se caractérise par sa survenue à un âge jeune, son importante taille tumorale et la fréquence de facteurs histopronostiques péjoratifs.

## Abstract (en)

Breast cancer is the most common cancer among tunisian women and worldwide. In Cape Bon, Tunisia, the anatomopathological features of this cancer have not been established in previously published studies. Knowledge about these features is needed for the cultural adaptation of prevention and health care systems in the region. The aim of our study was to determine the pathological profile of breast cancers in the only public health anatomic pathology regional laboratory. We conducted a retrospective descriptive study of 116 patients who were diagnosed with breast cancers in our laboratory over a 5-year period, from July 2010 to July 2015. Our study included 116 patients. The average age was 51 years. The mean histologic tumor size was 31 mm. The initial diagnosis was based on lumpectomy in 83% of the cases. Nonspecific invasive cancer was the most frequent histological type. SBR grade III was most prevalent. Lymphovascular invasion was detected in 33% of cases. Axillary lymph node dissection was performed in 72% of cases. Hormone receptors were positive in 73% of cases. Her2-Neu receptors were overexpressed in 19% of cases. The ki67 was ≥ 14% in 38% of cases. Luminal A was the most common molecular subtype. In Cap Bon region brest cancer is characterized by an early onset, a large tumor size and pejorative histoprognostic factors.

## Introduction

Le cancer du sein est le cancer le plus fréquent de la femme [1]. Dans le monde il est estimé qu'un million de femmes sont diagnostiquées d'un cancer du sein chaque année et plus que 400000 vont mourir de cette pathologie [2]. L'incidence du cancer du sein, sa mortalité et son coût sont considérables [3]. Dans les pays sous-développés et en voie de développement, en l'absence d'infrastructure et de moyens de dépistage, l'incidence et la mortalité du cancer du sein sont particulièrement en augmentation [2]. En Tunisie, à l'instar de la plupart des pays en voie de développement, l'incidence du cancer du sein chez la femme est en augmentation [4]. En effet d'après le registre des cancers 1999-2003, le risque de cancer du sein est en augmentation de 80% entre 1999 et 2024, soit en 25 ans [5]. Le cancer du sein en Tunisie se caractérise par la survenue à un âge jeune et par une taille tumorale importante, égale ou supérieure à 5 cm, au moment du diagnostic [6]. Dans la région du Cap Bon tunisien, les particularités anatomopathologiques du cancer du sein n'ont pas été précisées auparavant dans la littérature. Leur connaissance est nécessaire pour l'adaptation des systèmes de soins dans la région. L'objectif de cette étude unicentrique était de déterminer le profil macroscopique, microscopique, immunohistochimique et moléculaire ainsi que les particularités histo-pronostiques des carcinomes mammaires dans le service d'anatomie pathologique de l'Hôpital universitaire Mohamed Tahar Mâamouri de Nabeul, qui est le seul service d'anatomie pathologique publique du Cap Bon.

## Méthodes

*Type d'étude*: nous avons mené une étude descriptive rétrospective de 116 patientes ayant un carcinome mammaire in situ ou infiltrant diagnostiqué dans le service d'anatomie pathologique de l'Hôpital Mohamed Taher Mâamouri de Nabeul sur une période de 5 ans allant du 1er Juillet 2010 au 31 Juillet 2015.

*Critères d'inclusion*: ont été inclus tous les comptes rendus anatomopathologiques des cas de carcinomes mammaires infiltrants ou in situ de tous les groupes d'âges diagnostiqués sur microbiopsie ou sur pièce opératoire entre Juillet 2010 et Juillet 2015 dans le service d'anatomie pathologique de l'Hôpital Mohamed Taher Mâamouri.

*Critères de non inclusion*: n'ont pas été inclus: les tumeurs bénignes, les tumeurs malignes mésenchymateuses et lymphoïdes et les cas de récidive tumorale.

*Critères d'exclusion:* aucun cas n'a été exclu de notre série.

*Le recueil des données:* a été réalisé à partir des comptes rendus anatomopathologiques: les données cliniques (âge, côté du prélèvement, siège de la tumeur), les données macroscopiques (type du prélèvement, taille tumorale macroscopique), les données microscopiques (type histologique, taille tumorale histologique, nombre de tumeurs, index mitotique, grade histopronostique de Scarff-Bloom-Richardson (SBR) modifié par Elston et Ellis, emboles péri-tumoraux, association à un carcinome in situ, maladie de Paget du mamelon et statut ganglionnaire), les données immunohistochimiques (Récepteurs aux oestrogènes, récepteurs à la progestérone, surexpression de l'HER2-Neu et taux du Ki67) et les données moléculaires (luminal A, luminal B, HER2 et triple négatif).

*Etude statistique:* les données ont été analysées au moyen du logiciel SPSS version 19.0: Etude descriptive: Nous avons calculé des fréquences absolues et des fréquences relatives (pourcentages) pour les variables qualitatives. Nous avons calculé des moyennes, des médianes et des écarts-types et déterminé les valeurs extrêmes pour les variables quantitatives.

*Etude analytique:* les comparaisons de 2 moyennes sur séries indépendantes ont été effectuées au moyen du test t de Student pour séries indépendantes, et en cas de faibles effectifs par le test non paramétrique de Mann et Whitney. Les comparaisons de plusieurs (> 2) moyennes sur séries indépendantes ont été effectuées au moyen du test F de Snedecor d'analyse de la variance paramétrique (ANOVA à un facteur) et vérifiées en cas de faibles effectifs par le test H de Kruskall-Wallis d'analyse de la variance non paramétrique. En cas de différence significative, les comparaisons 2 à 2 ont été faites par la méthode de Bonferroni. Les comparaisons de pourcentages sur séries indépendantes ont été effectuées par le test du chi-deux de Pearson, et en cas de significativité au test du chi-deux et de non-validité de ce test et de comparaison de 2 pourcentages, par le test exact bilatéral de Fisher. Les liaisons entre 2 variables quantitatives ont été étudiées par le coefficient de corrélation de Pearson, et en cas de non-validité par le coefficient de corrélation des rangs de Spearman. Dans tous les tests, le seuil de signification statistique a été fixé à 0,05.

*Recherche bibliographique:* les mots clés ont été utilisés dans la recherche d'articles scientifiques. Les articles ont été identifiés dans les banques de données telles que Pubmed et science direct, transférés dans la base de données Zotero (version 3.0) et cités dans le texte via ce même logiciel.

## Résultats

**Figure 1:**
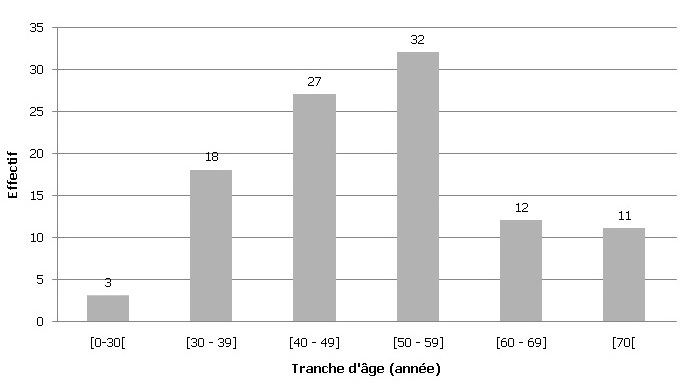
Répartition des patientes selon les tranches d’âge

**Sur le plan clinique:** L'âge moyen des patientes était de 51 ans ± 1,2 an, avec des extrêmes allant de 26 à 83 ans. L'âge médian était de 52 ans. Le pic de fréquence des carcinomes mammaires s'observait au cours de la cinquième décennie (Figure 1). La femme jeune (≤35 ans) représentait 10% des patientes. La femme âgée (≥70 ans) représentait également 10% des patientes. Nous avons analysé 147 tumeurs du sein diagnostiquées chez 116 femmes. Les tumeurs siégeaient au niveau du sein gauche dans 58% des cas et au niveau du sein droit dans 38%. Dans 3% des cas, l'atteinte était bilatérale: 1,5% de bilatéralité synchrone et 1,5% de bilatéralité métachrone. Le quadrant supéro-externe était le siège prépondérant, retrouvé dans 43% des tumeurs.

**Sur le plan macroscopique:** Les types de prélèvements reçus sont présentés dans le Tableau 1. Le diagnostic initial était posé dans la majorité des cas sur pièce de tumorectomie (Tableau 2). La taille tumorale macroscopique moyenne était de 32 mm avec une médiane de 28 mm et des extrêmes allant de 3 mm à 105 mm.

**Tableau 1:** Types de prélèvements

TYPE DU PRELEVEMENT	EFFECTIF
**Microbiopsie**	13
**Macrobiopsie**	3
**Pyramidectomie**	3
**Tumorectomie**	90
**Mastectomie avec curage axillaire**	59
**Recoupe**	19
**Curage axillaire**	17

**Tableau 2:** répartition des prélèvements à but diagnostique

TYPE DU PRELEVEMENT	Pourcentage (%)
**Microbiopsie**	12
**Macrobiopsie**	2,5
**Pyramidectomie**	2,5
**Tumorectomie**	83
**Total**	100

**Données de l'examen extemporané:** L'examen extemporané était réalisé pour 70 cas (59 pièces de tumorectomie, 2 macrobiopsies et 9 pièces de recoupe). Il était réalisé à but diagnostique dans 61 cas.

**Figure 2:**
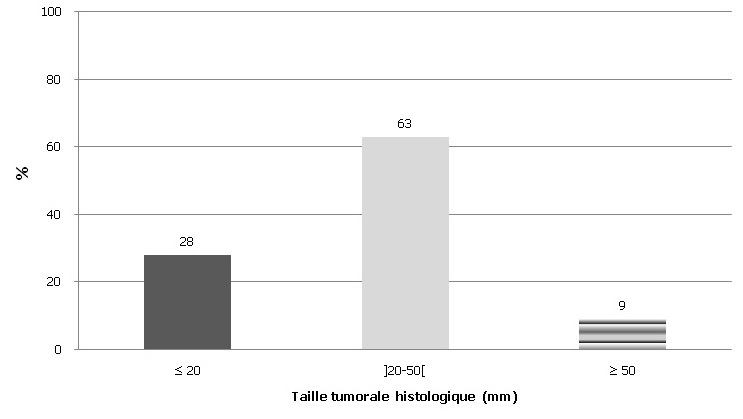
Répartition des tumeurs selon la taille tumorale histologique

**Sur le plan histologique:** La multifocalité était notée chez 15% des patientes. Le multicentrisme était observé dans 5% des cas. Le carcinome infiltrant de type non spécifique était le type histologique le plus fréquent, retrouvé 77% des cas. Il était suivi par le carcinome mixte et les carcinomes mucineux et papillaire. Le carcinome in situ pur a été retrouvé dans 1,4% des cas. La taille tumorale histologique variait de 1 à 105 mm avec une moyenne de 31 mm et une médiane à 27 mm. La majorité des tumeurs (64%) avait un grand axe entre 20 et 50 mm (Figure 2). Les tumeurs de grade histologique III étaient majoritaires représentant 41% des cas. Les grades II et I représentaient respectivement 38% et 21%. Les emboles péri-tumoraux étaient présents dans 33% des tumeurs. Une composante in situ était associée au carcinome infiltrant dans 36% des cas. Dans 69% de ces cas, il s'agissait de carcinome in situ de haut grade. Le plan profond était atteint dans 12% pièces de mastectomies. La maladie de Paget du mamelon était présente dans 23% des pièces de mastectomies. Les ganglions étaient positifs dans 72% pièces de curage axillaire. Le nombre de ganglions prélevés variait de 1 à 30 ganglions avec un nombre médian de 8 ganglions. Une atteinte métastatique d'au moins 10 ganglions axillaires était notée chez 21% patientes. Le statut ganglionnaire était significativement corrélé à la taille tumorale (p=0,039) avec un Odds ratio à 3. De plus, les emboles lympho-vasculaires étaient plus fréquents en cas d'atteinte ganglionnaire (p < 0,0001) avec un Odd ratio à 10,6.

**Figure 3:**
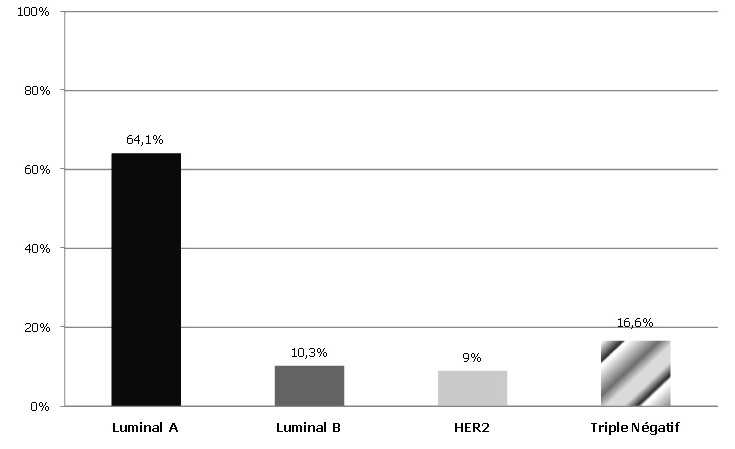
Répartition des tumeurs selon les classes moléculaires

**Sur le plan immunohistochimique:** Les récepteurs hormonaux étaient positifs dans 73% tumeurs. Les Récepteurs aux oestrogènes étaient exprimés dans 71% des tumeurs tandis que les récepteurs à la progestérone 64,4%. Une surexpression de l'HER2 (score3) était observée dans 19% tumeurs. Le Ki67 était ≥14% dans 38% des cas. Quant à la classification moléculaire, le phénotype luminal A était le plus fréquent (Figure 3).

## Discussion

Le cancer du sein est le plus fréquent des cancers féminins dans le monde [1]. Selon les séries du Globocan de l'agence internationale de recherche sur le cancer, le nombre de nouveaux cas pour l'année 2012 dans le monde est estimé à 1,7 millions [2]. L'incidence de ce cancer est plus élevée dans les pays développés, où elle est équivalente à deux fois et demie l'incidence constatée dans les pays en voie de développement [7]. En Tunisie, selon les données du registre des cancers du Nord Tunisie 2004-2006, le cancer du sein demeure le cancer féminin le plus fréquent, occupant largement la première place devant celui du col de l'utérus [4]. L'âge moyen de diagnostic de nos patientes est concordant avec les valeurs rapportées dans les autres séries tunisiennes variant entre 50 et 52,9 ans [4, 8, 9]. Il est également proche des valeurs rapportées dans les pays en voie de développement et les autres pays arabes où le cancer du sein est diagnostiqué à un âge moyen qui varie entre 46 et 48 ans [10–13]. L'âge moyen au moment du diagnostic varie fortement entre les pays en voie de développement et les pays développés où l'âge moyen au moment du diagnostic s'élève à 60 ans [14]. Ceci serait expliqué en partie par une répartition particulière de la pyramide des âges en Tunisie et dans les pays en voie de développement en général, en faveur d'une population plus jeune en comparaison avec les pays développés [15]. Dans notre série comme dans la littérature, le sein gauche est plus fréquemment atteint que le droit [16, 17]. Les taux de bilatéralité synchrone varient dans les séries publiées de 0,2 à 3,2% [18, 19]. Cette variabilité pourrait être expliquée d'une part par l'inconstante inclusion des carcinomes in situ dans la définition et d'autre part par une variabilité des définitions de la bilatéralité synchrone selon les auteurs. Dans notre série. Le diagnostic était rarement posé sur biopsie chirurgicale et microbiopsie. Ceci est attribué à l'inconstante disponibilité du personnel médical qualifié en matière de microbiopsie mammaire et des moyens matériels dans notre établissement. D'après les recommandations de l´Initiative mondiale pour la santé du sein (The Breast Health Global Initiative Resource-Stratified Matrix Guidelines) pour les pays à ressources limitées la microbiopsie mammaire est située dans le niveau 2 de la prise en charge diagnostique du cancer du sein). A défaut, la réalisation de biopsie chirurgicale devient absolument nécessaire [20]. Le siège prédominant de la tumeur chez nos patientes était le quadrant supéro-externe. Ceci est en accord avec plusieurs séries de la littérature [21, 22]. La multifocalité et le multicentrisme rapportés dans les études varient de 6 à 60%. Cette large variabilté est due à la différence des définitions adoptées, à l'inclusion ou l'exclusion de la maladie intra-canalaire et aux méthodes d'échantillonnage [23]. Le taux de carcinome in situ est de 3,3% dans le registre des cancers Nord-Tunisie 2004-2006 [4]. Cette différence par rapport à notre série peut être due au fait que les registres tunisiens concernent aussi bien le secteur publique que le secteur privé où le dépistage individuel est plus accessible. Dans les pays en développement, le taux de carcinome in situ varie entre 0,3% et 4,5% [10, 24]. Aux Etats-Unis, ce taux est évalué à 32,5% en 2004 [25]. L'accessibilité à la mammographie tant pour le dépistage que pour le diagnostic explique en partie cette nette augmentation [25]. Le carcinome mammaire dans notre série se caractérisait par un mauvais pronostic avec une taille tumorale histologique moyenne nettement supérieure à la taille tumorale histologique moyenne rapportée dans les pays occidentaux (25-26 mm) [26, 27], un grade histologique III majoritaire alors que dans les séries occidentales le II est prépondérant [28, 29], la fréquence élevée des emboles péri-tumoraux et de l'envahissement ganglionnaire ainsi que la fréquence de la négativité des récepteurs hormonaux et le taux élevé du Ki-67. Le taux de la surexpression de l'Her2 dans notre série était égal à celui rapporté dans une série du nord tunisien [22] mais plus bas que celui d'une étude du centre du pays [9]. Cela pourrait en partie être expliqué par le fait qu'on ne dispose pas des résultats des études moléculaires (CISH) faites pour nos patientes de score 2 d'HER2. La distribution de la classification moléculaire dans notre étude est comparable à celle d'autres études publiées en Tunisie [22] et en occident [30].

## Conclusion

Le carcinome mammaire dans la région du Cap Bon se caractérise par sa survenue à un âge jeune, son importante taille tumorale et la fréquence de facteurs histopronostiques péjoratifs. Nos résultats justifient le besoin de développer des programmes effectifs visant à prévenir et à diagnostiquer précocement le cancer du sein dans la région. L'organisation d'une stratégie de dépistage nationale de masse est nécessaire pour réduire la morbidité et la mortalité liées au cancer du sein.

### Etat des connaissances actuelle sur le sujet

- Une forte incidence dans le monde entier;
- Prédominance des formes agressives du cancer du sein en Afrique et les autres pays en voie de développement à cause du diagnostic tardif.

### Contribution de notre étude à la connaissance

- Ce travail éclaire une grande partie de la situation du cancer du sein dans une région de la Tunisie. D'ailleurs, notre travail est, à notre connaissance, le premier traitant du cancer du sein dans la région du Cap Bon.
